# Mining Resource Corridor development in Nigeria: critical considerations and actions for a diversified and sustainable economic future

**DOI:** 10.1007/s13563-022-00307-5

**Published:** 2022-02-21

**Authors:** Smith I. Azubuike, Susan Nakanwagi, Jaqueline Pinto

**Affiliations:** 1 School of Law, Queen’s University Belfast, University Road, Belfast, BT7 1NN Northern Ireland UK; 2 Centre for Energy, Petroleum and Mineral Law & Policy, University of Dundee, Scotland, DD1 4HN UK

**Keywords:** Mining Resource Corridors, Economic diversification, Nigeria, Community engagement, Infrastructural development, Sustainable future

## Abstract

The low crude oil prices in 2019 add momentum to Nigeria’s need for economic diversification as its foreign earnings come primarily from crude oil sales. As a result, Nigeria is seeking to explore other economic potentials, such as developing a Mining Resource Corridor (MRC) to reduce poverty and assist the country’s sustained growth and development. MRCs catalyse mineral extraction, transportation, processing, and infrastructural development and integrate other sectors of the economy, thus, creating jobs and promoting economic diversification and inclusive growth. However, achieving this requires drawing up an articulated delivery mechanism through a framework strategic implementation plan to attain a strategic diversification away from hydrocarbons to minerals. This article takes an applied research approach to examine the critical considerations and actions that Nigeria should take in developing an MRC to ensure a diverse and sustainable economic future. Additionally, it discusses the lessons Nigeria can learn from two corridors in Mozambique. This study notes that the success of a MRC is a function of, among other things, a skilled and adequately strengthened institution, enabling infrastructure and the participation of communities in the decision-making process of the corridor development. It also includes the support of all levels of government and possible assistance from development partners and donor agencies such as the World Bank.

## Introduction

The plunge in oil prices between 2018 and 2019 (US EIA 2020) and the COVID-19 pandemic induced fall in crude oil prices (Rizvi and Itani, 2021) are among the major drivers for the Federal Republic of Nigeria (Nigeria) to develop a Mining Resource Corridor (MRC). In 2020, global oil demand plunged when governments closed business operations and limited travel or imposed total economy lockdowns in response to the COVID-19 pandemic (Nakanwagi and Rukundo, 2020). The COVID-19 pandemic induced a great demand shock in the oil sector, resulting in the unprecedented market and worldwide economic collapse, including in Nigeria (Ozili, 2020).

This situation highlights the need for economic diversification. One way of achieving it in a mono-economy like Nigeria is by developing a MRC and upscaling access to its mineral resources. A MRC has the potential to assist Nigeria in diversifying the economy through mineral extraction, processing and transportation, infrastructural development, and possible sectoral integration and connection with other regional corridors. This could, in turn, create jobs and promote inclusive growth and development. For instance, since the mining sector is important to the country’s development objectives (MMSD, 2016), a MRC will aid the sector’s resurgence. Nigeria is endowed with various mineral resources, like gold, iron ore, coal, bitumen, baryte, lead–zinc, limestone, copper, lithium, and many other minerals and metals (Olade, 2019). These minerals provide options for economic diversification and sustainable development through a MRC. Previously, mining contributed a higher percentage of economic output before the sectorial dominance of the hydrocarbon sector in Nigeria in the 1950s and 1960s (Chete et al., 2017).

Developing a Resource Corridor (RC) requires an understanding of its operation to enable a diversified economy. A RC is an order of investments and actions to leverage a significant extractive industry investment in infrastructure, goods and services, into viable economic development and diversification along a specific geographic area (Jaffrin, 2013). RCs create development corridors alongside extractive-related infrastructure by linking investments through mines, ports, rails, and roads that catalyses ancillary economic activity (Cameron and Stanley, 2017). They also exist in various types and focus on transportation, agriculture, and mining. RCs require active collaboration, coordination, and integrated planning for effective implementation (Hope and Cox, 2015). Toledano (2015) notes that RCs present opportunities to connect communities and attract economic growth and sustainable development, mainly through the collateral impact on other sectors catalysed through access to resource infrastructure.

An analysis of the literature on RCs reveals that their development can result in social and environmental disruptions and community opposition when communities along the corridor routes are not engaged in the development process (Mtegha et al., 2012). They also can result in spatial exclusions, thus making it necessary to use a new mobility paradigm to avoid spatial exclusion, immobility, and uneven and conflicting mobilities in new corridor routes (Enns, 2018). Furthermore, as was the case with the China-Pakistan Economic Corridor, security challenges can halt the development process due to armed militia and terrorist groups (Ahmad et al., 2020). Given the difficulties highlighted, Nigeria requires a well-articulated delivery mechanism. This strategy must be drawn up and initiated through a corridor programme setting out the strategic plan and governance mechanism.

Further, security apparatuses must be in place to check extremist groups’ activities, especially in the northern part of Nigeria. Community engagement is also essential to prevent conflicts that can halt the development process. There is also a need for social and environmental impact assessments to determine the corridor’s viability vis-a-vis the sustainable development agenda. The full support of government at all levels and the political will to drive the corridor’s implementation is critical to its success, including infrastructural development and anchor projects. Funding options and development assistance from donor agencies and development partners are crucial aspects of corridor establishment in Nigeria.

Lessons from the successes and challenges of corridors in Mozambique, such as the Maputo Development Corridor (MDC), and the Nacala Corridor, can assist Nigeria in planning and implementing its MRC. This study chose Mozambique’s corridors because it houses some of the most developed corridors in Sub-Saharan Africa. Furthermore, these corridors are essential to Mozambique’s planning for various sectors and appear in its 2011–2020 Strategic Plan for the Development of the Agriculture Sector. Again, Mozambique has an established coal mining industry and a burgeoning hydrocarbon industry.

This article utilises an applied research approach to examine the critical considerations and actions that Nigeria can follow in developing a MRC. The “Developing a Resource Corridor” section presents an overview of MRCs and some discussions critical to RC development. The “Nigeria’s mining sector in focus” section provides a brief background to mining in Nigeria. The “Lessons from Resource Corridors in Mozambique” section evaluates the MRCs in Mozambique and highlights lessons that Nigeria can learn from them. The “Applying the lessons to the Nigerian context” section applies those lessons to the Nigerian situation, and the “Recommendations and conclusion” section sets out key recommendations for Nigeria to actualise its MRC development.

## Developing a Resource Corridor

### Resource Corridors: an overview and rationale for development

As a concept, RCs have gained significant traction in both developed, emerging, and developing climes (Jaffrin, 2013; Xu et al., 2019; Baxter et al., 2017). For example, the coal belt in England and the Ruhr Valley in Germany became industrialised in the eighteenth and nineteenth centuries through RCs and enhanced during the industrial revolution (Clark and Jacks, 2007; Popkin, 2020). RCs are fast becoming a means through which countries promote economic activities by opening up either transport, agricultural, industrial, trade, freight, mining, or other development corridors. Whatever the name, they all contribute towards economic development.

The World Bank (2011) describes a RC as “a sequence of investments and actions to leverage a large extractive industry investment in infrastructure, goods and services, into viable economic development and diversification along a specific geographic area”. This definition identifies that RCs benefit from existing extractive and other resources and investment to upscale economic diversification and development in an area. The description also shows the functional and physical aspects of a RC. The latter dimension relates, for instance, to transport routes that link regions of economic activity. The former relates to the institutions, plans, and framework that enhance the operations of a corridor.

A vital rationale for developing a RC is because it is an essential economic integration instrument and can open up markets and enlarge investment and trade (Bowland and Otto, 2012). RCs follow the Spatial Development Initiative (SDI) approach. RCs use economic growth opportunities to encourage private sector investments and sustainable development, primarily through the collateral impact on other sectors catalysed through access to the resource infrastructure. SDIs endorse infrastructure use and investment in specific geographical areas, thus encouraging linkages between local economies and overall competitiveness (Bastida, 2014). They also function on the substantial investment of public capital and resources, several stakeholders’ existence, actual economic potential, and private capital participation to achieve the common goal of economic growth and development (Bastida, 2014). As such, a government must consider critical aspects such as institutional capacities, funding and investment avenue, impacts on local communities, stakeholder engagement, security, and political will, in the development process.

RCs attract investments by identifying opportunities, providing infrastructure, and removing institutional and administrative hurdles that may exist (Shroder, 2014). The result is economic growth and job creation for local people. RCs play a vital role in facilitating access to ports and other trade routes that offer new global markets access. They optimise the use of hard infrastructure built by or with the support of an anchor project to support other sectors through further investments, such as building feeder roads to help develop agricultural projects.

Cameron (2016) notes that a RC’s objective should be to respond to a country’s needs from an extractive perspective. Thus, it should enable the use of significant commercial extractive investments and their need for infrastructure, goods, and services as an anchor for more wide-ranging economic growth and diversification. RC thrives on transport investments such as port, rail, and road, stimulating additional economic activity, thereby creating corridors alongside extractive-related infrastructure. This perspective entails investment in hard and soft infrastructure that will facilitate corridor activities and develop local content.

From an investment perspective, RCs create the pooling of resources and provide the opportunity to develop cross-border organisations to promote investment, energy utilities, transport businesses, and border administration (Oxfam, 2017). Thus, they encourage economic linkages and cross-border activities between neighbouring countries, open up many remote areas to modern agriculture and facilitate economic growth (Weng et al., 2013). In their work on “Resource Corridor Experiences in Sub-Saharan Africa”, Mtegha et al. (2012) highlight the essence of an anchor project regarding corridor establishment. They also emphasise that an active project manager and multilateral agreements are vital, especially for establishing a cross-border RC. Additionally, RCs thrive on regulatory reforms, investment promotion initiatives, a robust institution for success, and stakeholder participation (Shroder, 2013).

In choosing a RC location, it is essential to consider how the area will allow for and integrate active, practicable, or potential economic growth and link other substantive economic nodes that have the potential for growth between them (Srivastava, 2011). Additionally, it is critical to understand the variations in opportunity associated with particular minerals. For example, the mining of coal would necessitate the establishment of railways. However, the extraction of gold would require the creation of roads and access to vast amounts of water. Other key factors for consideration when selecting the RC location include the existing routes and corridors, anchor projects and their locations, potential policy and regulatory constraints, and the capacity to promote, integrate, and encourage trade. Further, a country should evaluate the RC’s impact on the communities along the route to alleviate their plight. They should also look into the potential for small, medium, and micro-enterprise development via existing agricultural supply chains (Mtegha et al., 2012).

RCs are not new in Africa. The Maputo Development Corridor (MDC), Mtwara Development Corridor Tanzania, the Lamu Port South Sudan Ethiopia Transport (LAPSSET) Corridor, the Nacala Corridor in Mozambique, and other corridors criss-crossing Africa and opening areas for investment exist (Laurance et al., 2015). Officially launched in 1996, the MDC is a transportation network that includes roads, rail, border posts, ports, and terminals. The Corridor passes through Southern Africa’s most industrialised landlocked provinces, including Mpumalanga, Gauteng, and Limpopo (Tevera and Chimhowu, 2018). The Mtwara Development Corridor, on the other hand, spans southern Tanzania, northern Mozambique, northern and central Malawi, and eastern and northern Zambia (Weng et al., 2013). Its development followed a memorandum of understanding by the respective country heads of state in 2004. It currently has road and rail links, bridges, and ports.

As part of the 2008 Kenya’s Vision 2030, the LAPSSET Corridor Project aims to interconnect Kenya, Ethiopia, and South Sudan in East Africa, with Kenya pioneering the Project’s implementation (Lesutis, 2020). The project is comprised of two critical components: a 500-m-wide infrastructure corridor through which roads, railways, pipelines, power transmission, and other projects, including the proposed Lamu Port with thirty-two deep-sea berths at Manda Bay, will pass; and a 50-km-long economic corridor on either side of the infrastructure corridor. Further, the Nacala Corridor Railway and Port project in Mozambique and Malawi is intended to facilitate the development and exploration of Vale Mozambique’s coal deposits in Moatize. The railway construction was started in 2011 and completed in 2016.

The successes and challenges of existing corridors in Africa act as a signpost for planned and emerging corridors. For instance, the MDC highlights the importance of a political will and political champions and the need for a long-term strategy for economic development and integration, which addresses the envisioned role of development corridors, and how to realise it (Bowland and Otto, 2018).

Within the African context, community engagement constitutes a critical aspect during corridor development. The reason is that community livelihood and the environment are not free from the possible impacts of RC development. Thus, engaging with affected communities provide pathways for them to develop sustainably and avoid conflicts in the development process. Illustrative of this importance is the LAPSSET Corridor, which witnessed an increased risk of disruption, and the potential for community grievances to derail the corridor project due to the absence of community engagement (Baxter et al., 2017). The MDC also provides another example. It had a deficiency in community engagement at the planning stage and a lack of political will in creating the local capacity to manage the MDC process or involve the broader community and local levels of governance (Roodt, 2008). Enns (2018) writes that RC development in Africa should consider novel patterns of spatial inclusion and exclusion and mobility and immobility along new corridor routes. The essence is to present evidence that supports a win–win narrative that is presently attached to Africa’s corridor agenda.

Security threats, terrorism, and civil disturbances in a country are essential aspects of the RC debate. This issue can derail or hinder RC establishment. For example, Ahmad et al. (2020) observe that security and terrorism in Pakistan, among others, constitute an impediment to achieving the benefits of the China–Pakistan Economic Corridor (CPEC). The threats are both internal and external, arising from terrorism and extremism in the two countries. Extremist groups exist from Xinjiang to Gwadar, consisting of the East Turkestan Islamic Movement, Tehreek-e-Taliban Pakistan, Lashkar e-Tayyiba, Lashkar e-Jhangvi, Daesh, and Balochistan Liberation Front. These groups and the militant wings of some political parties tried to stop the CPEC project (Ahmad et al., 2020; Ibrar et al., 2016).

Although RCs can support economic growth, enhance national infrastructure, boost agriculture, increase exports, and improve economic integration, they present social and environmental challenges. RCs can result in uneven development impacts, further marginalise the poor, threaten biodiversity, and be vulnerable to climate change. In estimating the ecological costs of corridors, Laurance et al. (2015) note that corridors can result in severe and mostly irreversible environmental changes and should only proceed if the government have deployed mitigation and protection measures.

Development partners and spatial planners have widely discussed and debated the potential for RCs, anchored by an extractives project, to support sustainable development (Baxter et al., 2017).

There is a general agreement that RCs present a platform to ensure the distribution of benefits from a particular (mining) project by connecting other parts of the economy. Interest in RCs, such as the New Partnership for African Development collaboration with Nigeria, lends credence to this fact, albeit this potential has not been fully realised in other RCs. Baxter et al. (2017) note that some corridors’ inability to attain full potential is due to poor planning and lack of engagement with project-affected communities, often resulting in limited achievement. They also highlight weak institutional capacities and the absence of an integrated approach to planning as a challenge. A case in point is the North–South Corridor connecting the Democratic Republic of Congo and Zambia. Also, the lack of collaboration between various stakeholders such as governments, civil society, donor agencies, the private sector, and communities impedes the projects.

To realise the transformational benefits of a corridor initiative and diversify an economy, develop a nation, and reduce poverty, a government’s commitment and political will to implement the corridor initiative are crucial. As Nigeria plans to establish a MRC, this article points to the critical issues that Nigeria should consider and the possible implications and problems that may arise in the implementation process.

### The relationship between MRC, economic linkages, and sustainable development

A MRC uses significant extractive industry projects as an anchor in order to create economic development and diversification along a specific geographic area and creates linkages with other sectors such as agriculture and commerce (Atta-Mensah et al. 2021). MRCs create spatial ties through economic and infrastructural development, an essential link to all ancillary opportunities in a corridor. Fiscal linkages emerge from MRCs through the efficient use of resource rent. Knowledge linkages are created through technology and capacity development, allowing industries to flourish without any necessary connection to an anchor project and is a precondition for all other ties.

MRCs create backward linkages by allowing the flow of goods and services that are diversified and flexible and can last beyond resource depletion. Forward linkages arise from MRCs through the beneficiation of products (Wasonga and Smith, 2017). However, it should not be relied upon entirely as it can be subdued in the long term by the finite resources of an anchor project. MRCs also catalyse economic diversification by contributing to agricultural growth and food security and providing opportunities for broad economic opportunities (Kuhlmann et al., 2011). Broadly, MRCs assist in creating economies of scale, especially in developing country markets that cannot attract the private investments necessary to increase exports and eventually attain the objective of poverty alleviation.

MRCs constitute natural markets for products; they can be used by people, businesses, and the entire country. MRCs can also be used as an innovative approach to foster the growth of the agricultural sector by facilitating the movement of farm produce, goods, and services for the farmers (Kuhlmann et al., 2011). The existence of a MRC also enhances a country’s ability to trade regionally through improved ports and rail networks.

In the context of sustainable development, MRCs play an essential role. Although the concept of sustainable development has various perspectives, broadly, it involves improved environmental protection with more significant socio-economic growth and development (Hilson and Murck, 2000). It is a growth path that allows present and future generations to achieve their goals. MRCs support sustainable development through the linkages it creates with other sectors of the economy. A single mining operation can contribute to sustainable development from mineral exploration to its likely and certain closure. However, sustainable development is advanced beyond just one project by utilising a mining operation as an anchor to create diversified economic opportunities and diverse industries. It also creates an opportunity for socio-economic growth further than the anchor project itself.

The transport industry quickly arises from a MRC with accompanying jobs and linkages to trade and agriculture. The opportunities to develop it will be specific to different MRCs that can bring investment to areas where otherwise no investment would flow and create routes for transporting goods. MRCs enable the prioritisation and promotion of infrastructure and investment in other sectors such as agriculture along the corridor’s designated route, thereby catalysing trade, further investment, and optimising the use of existing and newly constructed infrastructure (Krchnak et al., 2011). MRCs also offer communities a sustainable development platform as socio-economic, environmental impacts, and community needs will be part of the MRC development planning process, thus enabling meaningful engagement with communities during the corridor development process and preventing any adverse effects on their livelihood through effective planning to create sustainable businesses and developing socio-economic growth skills.

## Nigeria’s mining sector in focus

### Mining in Nigeria

Mining in Nigeria dates back to 1902 when organised mining in its exploration stage started with the then Secretary of State for Colonies commissioning mineral surveys of the Southern and Northern Protectorates (MMSD, 2016). The first record of a mined mineral was tin ore by the Royal Niger Company in 1905 (Hodder, 1959). Through the years, several minerals, like coal, gold, bitumen, baryte, lead–zinc, and limestone, have been extracted by both artisanal and industrial miners (Nigerian Bureau of Statistics, 2015). The period has also experienced the enactment of an indigenisation decree causing large-scale withdrawal of foreign investment in the industry and a downturn in production (Ministry of Mines and Steel Development (MMSD), 2016). Thus, the bulk of mining operations by the private sector rested on small-scale indigenous miners’ shoulders, leading to production decline, particularly in the metallic minerals starting in the late 1970s.

Despite several strategic minerals (Gabriel, 2015) and government reforms, the mining sector still struggles to reach its full potential. One reason for this stems from the neglect of mining for the high revenue oil sector. Such neglect has seen the absence of a mining strategy and a corridor to enhance mineral activities which could be said to contribute to the mining sector struggling to reach its full potential in that, prior to 2016, there has been very little formal policy focus on the strategic development of the mining industry or economic linkages which could flow from such industry. Following the adoption of the Roadmap for the Growth and Development of the Nigerian Mining Industry in 2016 (Mining Roadmap, 2016), a MRC can stimulate the mineral sector and assist in economic diversification and growth.

Mining, agriculture, and petroleum sectors are essential to the Nigerian economy. However, the latter has received more attention than the former two sectors as the nation’s economy thrives on proceeds from crude oil sales, accounting for the low contribution of mining to the nation’s GDP (Olalekan et al., 2016). Insufficient exploration investment and the inability to harness its wealth creation capacities led to gross underperformance in the sector and, consequently, a loss of economic opportunities (Arome and Enejoh, 2019).

With renewed investor interest in mining and its immense mineral deposits, Nigeria can become a market figure in the mining sector (Liedtke, 2020). The Mining Roadmap, 2016, highlights the potential for an increase in the sector’s contribution to the country’s GDP, thus supporting forecasts that a concentrated exploration of Nigeria’s abundant minerals may exceed their oil wealth in the long term (Oxford Business Group, 2019).

Furthermore, the essence of the National Minerals and Metals Policy is to promote a new legislative framework that encourages private sector-led growth and development of the sector (KPMG, 2017). This regulatory shift could translate to increased public and private sector investment, more employment creation for the citizenry, and overall economic and financial stability for the economy if implemented correctly. A MRC will calibrate these new investment interests to promote investment in the mining sector, diversify the economy away from oil and gas, and preserve the much-needed forex spent on steel imports annually. Developing a MRC will also create wealth, promote alternative sources of public revenue, and lift millions out of poverty (Oxford Business Group, 2019).

To lessen the post-COVID-19 economic shocks, the World Bank has advised Nigeria to develop the mining sector instead of waiting on oil prices to rebound (Francis, 2020). Thus, a MRC is a platform to boost mineral activities and link Nigeria with existing corridors such as the Lagos-Kano-Jibiya corridor to Niger and the Abidjan-Lagos-Dakar corridor along the coast (Saana Consulting, 2015). However, since MRCs function on extractive industry anchors, those anchors will need to be identified and the possibility of economic diversification evaluated before connecting with other corridors.

### Mining Resource Corridors development in Nigeria

#### What is the rationale for establishing MRCs?

The plunge in oil prices in 2019 and the COVID-19-induced lockdown affected mono-economies like Nigeria. Thus, Nigeria is seeking alternative means to create wealth, boost mining activities, revive ailing industries, and diversify the economy by developing a MRC. A RC attract multiple stakeholders and provide economic opportunities for remote and isolated communities and towns located away from urban centres to develop. Nigeria understands that a MRC usually attracts other services and amenities like water, power supply, and transport infrastructure. These infrastructures bring significant benefits to communities and open them up to new trading opportunities. They also assist in upscaling artisanal miners’ engagement in mineral extraction.

Most of Nigeria’s mineral resources are found in remote and rural areas. For example, gold deposits are located in the country’s northern region, in Maru, Osun state, Malele, Okolom-Dongondaji, Anka, and Iperindo. Iron deposits are mainly found in the remote regions in Itakpe in the Kogi State. Also, gemstones like ruby, emerald, and sapphire are scattered in several remote areas of the country, for instance, Kaduna, Bach, and Plateau states. The map below shows the distribution of the vast mineral resources in Nigeria.

**Fig. 1:**
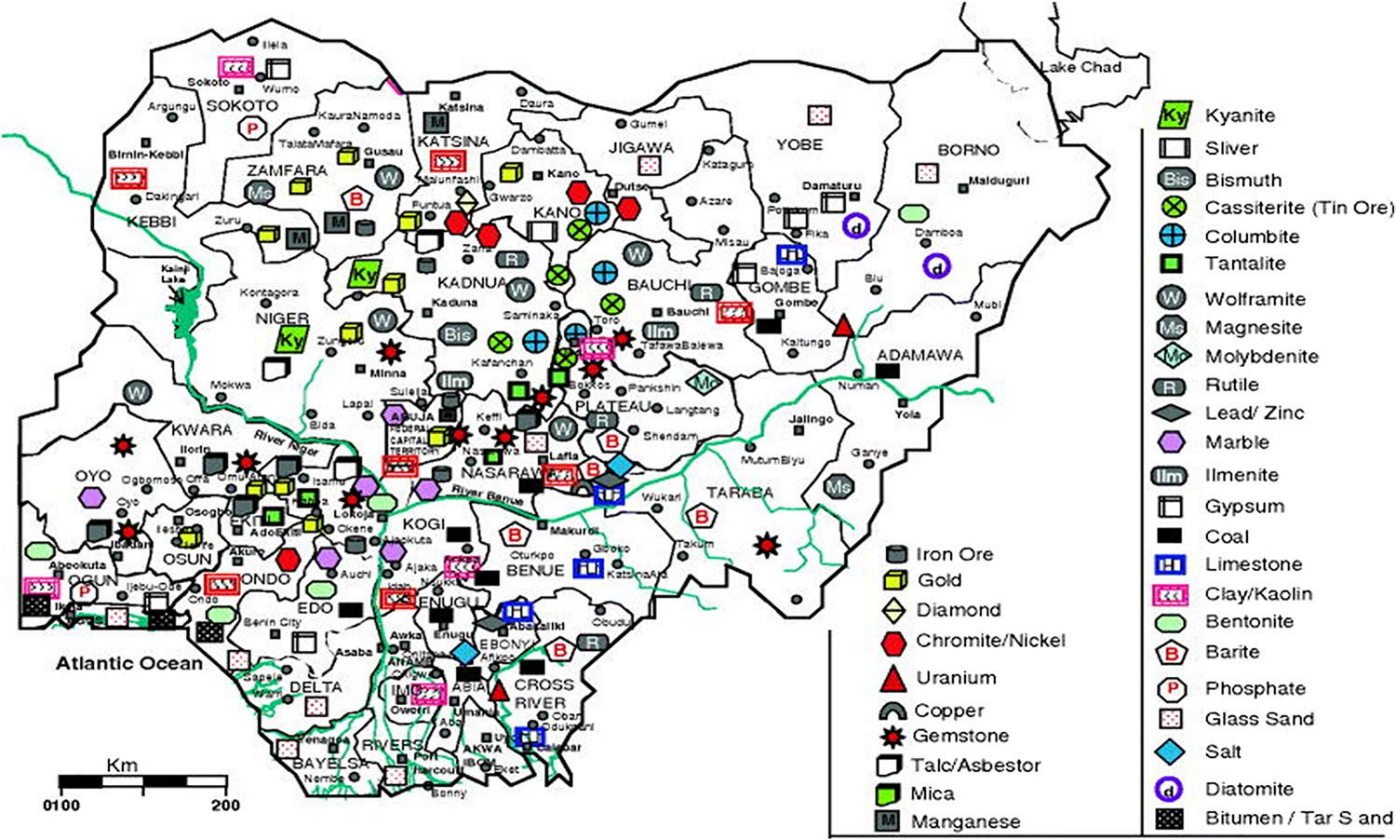
Map showing the mineral occurrences of Nigeria. Source: Obaje (2009)

The map above (Fig. 1) shows the vast array of mineral resources distributed across the country. With abundant minerals located in rural areas, a MRC in Nigeria will galvanise investment interest and boost the rural economy for sustained growth, thus promoting infrastructural development around the mining communities and creating linkages for other economic activities. Ramdoo (2015) notes that governments and regions must optimise the many types of links, including fiscal, production, and consumption linkages within and outside the extractive industry, to ensure the success of resource-based industrialisation. However, there is no one-size-fits-all solution to the issues faced by resource-rich countries in optimising their extractive industries. Therefore, there is a need for appropriate policy implementation measures from all stakeholders like the government, private sector actors, and global and regional actors.

Another rationale is that a MRC promotes growth, facilitates investment borrowing, and enables mining investors and communities to benefit from the venture. This means that Nigeria can boost agriculture in areas where the corridors exist as local farmers transport their produce via rail or road transport. Nigeria will also leverage the extractives sector to multiply jobs in the supply chain. Thus, enabling downstream development (e.g., smelting, refining, and beneficiation) and in related industries by seeking public and private sector synergies to prioritise investments. An example is the upscaling activities in the Ajaokuta and Aladja steel mill in Kogi State, Nigeria.

Developing a MRC is part of the broad objective of Nigeria to mainstream the Roadmap for the Growth and Development of the Nigerian Mining Industry developed in 2016. A MRC will serve as an economic conduit and amplify the growth opportunities, primarily as Nigeria depends on oil revenue for economic sustenance. Currently, mining contributes only 0.3% to the country’s GDP, but developing a MRC will upscale the sector’s contribution to national GDP. It will also create a synergy with other economic sectors and promote local content as rural communities participate in corridor activities. A case in point is Zamfara State which has the highest gold deposits in Nigeria. A MRC will facilitate legitimate mineral trade and ensure that both miners in Zamfara State and the country do not lose out on the reward of considerable natural assets in the marketplace and resource tax.

#### Developing a MRC in Nigeria

A fundamental step to developing a MRC is setting out a corridor programme, determining its objective and preparing for hard and soft infrastructural investments. Clarity of purpose will drive the attainment of the aim. The corridors must be purposeful and strategically located mainly as mining is site-specific. Some knowledge of the local area and a decisive approach to the corridor is essential.

For MRCs to be viable, they must make economic sense through encompassing actual or potential economic growth. The development of a corridor does not create economic strength as it channels, focuses, and amplifies economic growth potential. Thus, the significance of a MRC lies in its connection and passage through places of economic value. A corridor linking two substantive nodes but with no potential for growth in between is of limited benefit (Srivastava, 2011).

There is a need for initial construction of road, rail, and other infrastructure or upgrading existing infrastructures. The aim is to enable the movement of goods and people from one point to another. In the case of Nigeria, some of this is already happening. For example, the country is keen on developing the Abuja Gateway Airport. It has also invested heavily in the Lagos-Ibadan rail and the 1,400-km Lagos-Calabar railway, estimated at approximately $11 billion, and connects the western and eastern regions.

Further, Nigeria has a public–private partnership (PPP) approach to finance the country’s infrastructure development (Itu and Kenigua, 2021). The PPP infrastructure development in Nigeria is regulated by the Infrastructure Concession Regulatory Commission (ICRC). The Commission is currently implementing approximately 76 PPP projects, including the Bonny Deep Water Port Project (Itu and Kenigua, 2021). Therefore, the MRC developers should also enlarge existing corridors, and the construction should consider local, provincial, and national actors’ in the development plans. This would include urbanisation and agricultural nodes, and it will enhance the climate for business, such as providing small loans for business development.

It is necessary to determine if the MRC will be domestic or linked to other regional corridors to facilitate trade opportunities with other countries. The latter case requires bilateral or multilateral agreements. The economic potential of a mineral in an area is crucial to corridor development. It means that a large concentration of minerals will require the critical infrastructure to facilitate the transportation of minerals and give investors and other stakeholders access to the mineral market.

The government of Nigeria must evaluate existing and proposed routes in the corridor development process.

### Critical considerations

#### Who will fund the MRC?

Two funding streams are critical to establishing a MRC—funding for project development and funding for maintenance. An option is to invite mining companies to fund the project through PPP or direct government funding for the former. PPPs are a common way of financing MRCs, albeit a combination of donor funding supported by counterpart government funding exists. Donor agencies such as the World Bank could support corridor development, and recipient governments can be responsible for providing all recurrent downstream financing for scheduled routine and period maintenance interventions during the infrastructure’s operational life (Hope and Cox, 2015).

The MDC strategy was to use tolls paid by users; in the Mumbai-Delhi Dedicated Freight Corridor, freight handling charges were used, and in the Maputo Port, through docking, loading, and unloading charges (Bowland and Otto, 2012). For privately operated infrastructure, some of the generated revenue will be set aside for maintenance activities. In a government-managed project, it is necessary to establish a dedicated fund derived from end-users. In such cases, these funds should be devoted to maintaining the infrastructure from which the funds are acquired, rather than in governments’ general revenue for allocation to other sectors.

The maintenance of infrastructure requires dedicated organisations with a well-trained and experienced team. In turn, this requires strengthening existing agencies or setting up personalised organisations, both private and public. For instance, the mandate of the ICRC in Nigeria could be extended to include infrastructure maintenance oversight. Properly maintained infrastructure will give governments, communities, and the private sector the necessary confidence to continue to invest in economic activities whose growth is dependent on the hard infrastructure. It may also be essential to consider the cost that the country will face if a private extractive company was solely involved in the project and the security of tenure for a mining company funding the corridor development.

#### How will the MRC be implemented?

The implementation of a MRC is essential to its realisation. Effective implementation will depend on the type, stage of evolution, and range of stakeholders involved at any development phase. The MRC should divide the investment into critical steps such as infrastructure, livelihoods, governance, environmental impact, and social issues. In addition, the development plan should spread across timelines. All development takes a certain amount of time—short-term, medium-term, and long-term—whether for a mine to come online, build a railroad, or train personnel (Shroder, 2013).

In the development stage, the MRC should set out its specific focus in an area or region. For instance, will the MRC focus on gold or coal production and transportation? Each of these corridors should have a long-term option to further increase prospects to other regions. Ultimately, the economics of a corridor drives its development, and the economic viability of its transport route depends on feasibility studies and the hard infrastructure of one or more transport modes. As more freight and people move along the corridor, the soft infrastructure (logistics and institutions) needs to improve to maintain or increase efficiency. Efficient corridor operations encourage further economic activity, leading to new investment, and, ultimately, the corridor evolves into a commercial corridor.

Nigeria should set a corridor’s objective using relevant laws, policies, and strategies for sustainable development and protect and encourage investment. In implementing a governance initiative like the Maputo Corridor Logistics Initiative (MCLI), it is necessary to facilitate activities and address issues that could impact smooth operations (Bowland and Otto, 2012). The MDC’s success is attributable to having a centralised governance structure in the MCLI platform (Rasagam et al., 2014). Transport and logistics are also essential in implementing a MRC to enable cooperation and networking among investors.

#### How will the MRC be monitored and governed?

The Extractive Industry Value Chain (EIVC), promoted by the World Bank, provides a framework for monitoring corridors (World Bank, 2016). The EIVC is a framework for coordinating and integrating economic interactions across broad public and private collaborations. It incorporates the award of licences and contracts, monitoring operations, enforcing environmental protection and social mitigation requirements, collecting taxes, soundly distributing revenue, and implementing sustainable development policies.

Monitoring could be enforced by seeking to know if the corridor’s strategy ensures that bids are invited from interested investors and resource development contracts awarded to companies that submitted winning bids. A clear legal, fiscal, institutional, and contractual regime must be in place.

The selection criteria must be transparent and communities’ interests considered with informed, meaningful consultation from vulnerable groups, including women and youth representatives. It could also seek to examine technical and environmental regulations to ensure that they meet acceptable practice standards and that government agencies enforce the law.

Monitoring extends to reviewing institutions’ administrative and auditing capacity and ensuring that government revenues are deposited into a treasury and published. The monitoring process also encompasses general revenue management, allocation activities, and implementation of sustainable development goals (Alba, 2009). Through monitoring, the government can enforce compliance with the corridor rules and regulate and monitor operations, collect taxes and royalties, manage and allocate revenue, and finally, implement sustainable development policies. This monitoring mechanism has been applied successfully by numerous corridors and emerging market nations (Shroder, 2013).

Legislation should set out the governance structure and process for ease of reference and implementation. It should provide for a coordinator/manager for each of the corridors to enable efficient execution and include stakeholders from both the public and private sector, non-profit organisations, and community representatives to allow for a broad discussion on the desired future of the MRC. The policy and legislative regime should focus on the activities of the MRC and what it needs to achieve (Öberg et al., 2016). Where a board of directors and an executive committee is necessary, it should consist of executive and non-executive members responsible for promoting the corridor’s objectives. They should also provide the policy direction; monitor, implement, and operate structures, finances, and administration; and approve the corridor’s operating and capital budgets. The executive committee could consist of members of the Board of Directors. A similar governance structure exists in the MDC through the MCLI (Fouad, 2006).

#### Where will the MRC be?

**Fig. 2:**
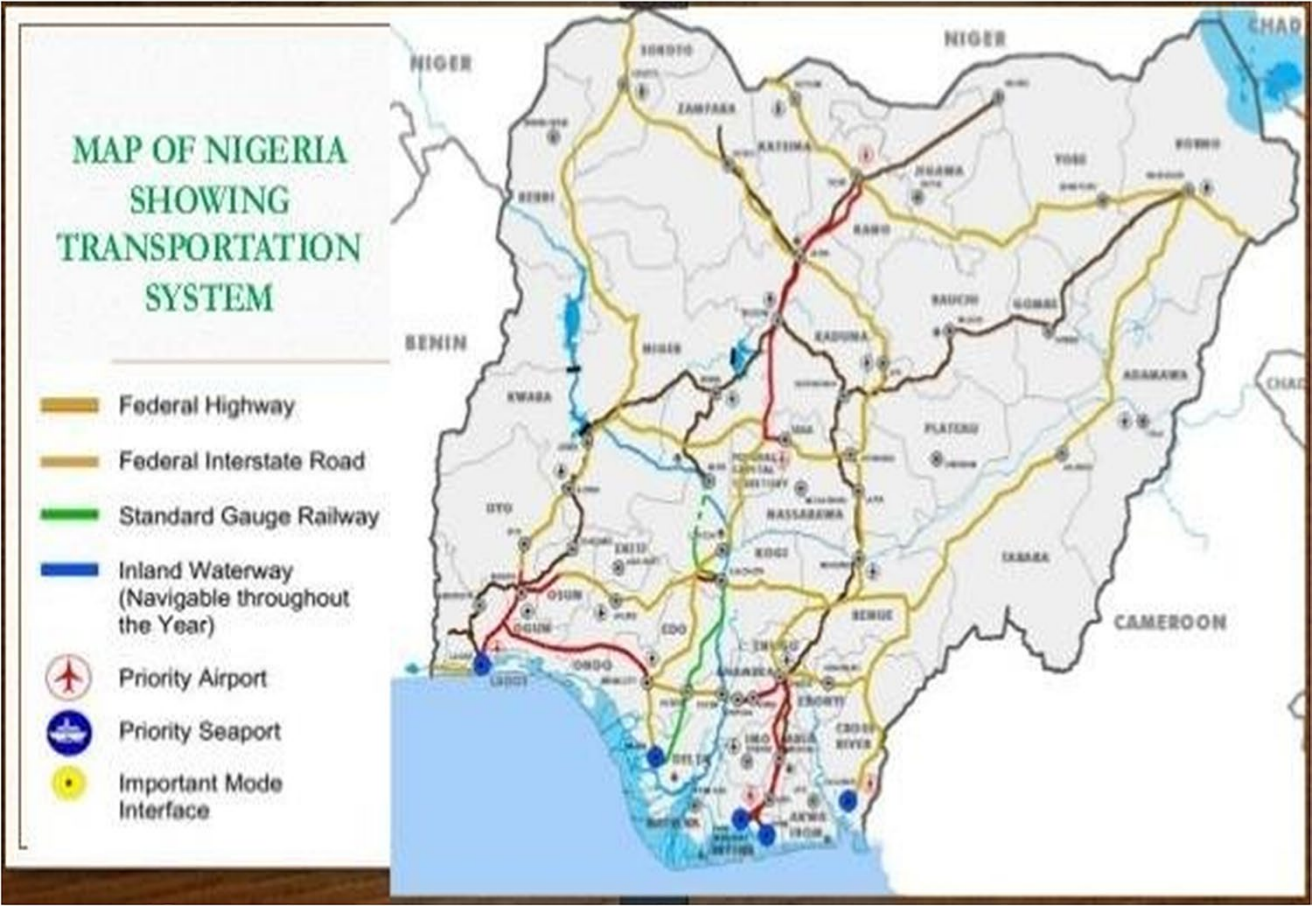
Showing the existing transport system in Nigeria. Source: Adanikin and Oyedepo (2017)

The decision concerning the location of a MRC is a function of the volume of minerals in an area, pre-existing transport facilities (as shown in Fig. 2), and other infrastructure that can enhance the corridor’s activities and promote resource trade and possible linkages with other regions.

The figure above shows that most of the transport facilities are concentrated along the urban populated and industrial areas, for example, the federal highways and federal interstate roads. The standard gauge railway also only covers a short area. However, these existing systems provide a fertile ground for expanding the transport network, especially the MDCs to cater to the rural and remote mining areas.

As Table 1 shows, there is a considerable concentration of minerals in the remote areas in Nigeria. For example, gold is found in the Kebbi, Zamfara, Niger, Kwara, and Kaduna axis, with smaller quantities scattered in other parts of the country (MMSD, 2021). There is also a large mass of iron ore in Kogi and Abuja and Kaduna, Katsina, and Bauchi. In addition, there are large bitumen deposits in Ogun, Ondo, Edo, Anambra, and Lagos States. Furthermore, Enugu is known as the Coal City, and there are coal deposits in Ebonyi, Anambra, Kogi, and Benue States. In addition, there are other strategic mineral deposits in the country, with oil and gas abundantly located in Nigeria’s Niger Delta (Emoyan et al., 2008).

**Table 1:** Sowing the location of selected minerals in Nigeria

Mineral resource	State(s) in which mineral is found
Gold	Kogi, Kaduna, Niger, Kebbi, Osun, Zamfara, Kaduna, Kwara
Tin	Kano, Bauchi, Kaduna, Kebbi, Plateau, Ekiti, Cross River, Nassarawa
Manganese	Katsina, Kaduna, Kebbi, Zamfara, Niger, Cross River
Copper	Plateau, Nassarawa, Bauchi, Kano, Zamfara, Yobe
Titanium	Cross River, Bauchi, Kaduna, Nassarawa, Katsina
Iron	Nassarawa, Kaduna, Zamfara, Enugu, Kogi, Oyo, Kebbi, Katsina
Gemstones	Kaduna, Bauchi, Nassarawa, Ogun, Plateau, Kogi, Niger, Taraba, Ogun, Oyo
Tungsten	Kaduna, Bauchi, Nassarawa, Plateau, Cross River, Kano, Niger
Chromium	Katsina, Kogi, Zamfara, Kaduna
Zinc	Zamfara, Kaduna, Cross River, Benue, Plateau, Nassarawa, Ebonyi
Lithium	Nassarawa, Zamfara, Ekiti, Niger, Kaduna, Nasarawa
Gypsum	Edo, Ogun, Sokoto, Adamawa, Yobe, Gombe, Ebonyi
Nickel	Kaduna

The Lagos-Kano rail line is being rehabilitated with new connections to facilitate linkages with other rural and urban areas (Ezeobi, 2019). There is also the Warri-Itakpe-Ajaokuta rail line, with a rail line proposed to connect the Abuja-Kaduna-Kano route. The Itakpe-Ajaokuta-Warri rail line links Warri in Delta State to Ajaokuta in Kogi State (Vanguard, 2020). The signing of a new contract for constructing a standard gauge railway from Abuja linking Warri from Itakpe with an extension to Warri port (Burroughs, 2019) presents opportunities for developing critical infrastructure.

Nigeria can establish a gold corridor, an iron ore corridor, a bitumen corridor, a coal corridor, or a central corridor linking to all these regions. Where the development of multiple MRCs is possible, Niger State should be the site of the gold corridor, Kogi State for iron ore, Enugu State for coal, and Lagos State for bitumen. These corridors will facilitate the transportation of minerals from extraction to processing points, primarily as transport and other infrastructures exist in these states. Ramdoo (2015) pointed out the lack of proper transportation infrastructure in the regional competitiveness of the cement industry, even though the government adopted a “backward integration policy” to facilitate its growth. However, where the odds favour a central corridor, Ajaokuta in Kogi State should serve as the country’s central corridor due to its steel rolling mill. It also connects the Lagos-Abuja-Kano rail line that can link the Lagos-Dakar transport route. The planned revival of the steel plant also gives Ajaokuta an advantage (CSL Stockbrokers, 2020).

### Potential benefits of MRC in Nigeria

From a gender perspective, developing a MRC in Nigeria will allow more women to be involved in the mining sector. Apart from being appointed as members of the corridors’ board, women will participate in mineral extraction as artisanal miners and other commercial activities that come with a corridor. In Nigeria, women have been confined to salt and sand mining to care for their families’ economic needs (Gender Resource Facility, 2016) and participate less in core mining activities due to occupational exclusion. This situation arises from cultural and religious beliefs and stereotypes. Thus, a MRC presents an opportunity for gender scalability in the planning and decision-making processes during the various stages of development.

With the Nigerian government’s directive that mineral operators should secure Community Development Agreements (CDA) (Sumaina, 2018), such agreements with operators can enhance community development in other respects and promote sustainable development. For example, the MRC can promote infrastructural development and agriculture as farmers convey their produce using rail or road transport. Furthermore, the benefits of employment and large-scale extractive and corridor activities flow to local communities through the market, fiscal mechanisms, and the environment.

### Lessons from Resource Corridors in Mozambique

#### The Maputo Development Corridor (MDC)

The MDC is one of the well-known corridors in Africa and is therefore instructive and serves as an example from which Nigeria can learn lessons. The MDC is a transport corridor connecting the Gauteng Province in South Africa to Maputo’s port in Mozambique. It aims to establish developmental cooperation between these two key areas and “unlocks” the landlocked provinces of Mpumalanga and Limpopo in South Africa and runs through landlocked Swaziland, situated within South Africa’s borders (Bowland and Otto, 2012; Cohen, 2008).

The MDC is arguably one of the most remarkable corridor initiatives, and its efficient organisation and success in PPPs have made it a model example despite having experienced challenges. One of its first and arguably most notable successes are the vital role it has played in the broader, regional initiative of linking the Atlantic and Indian Oceans through the Trans-Kalahari and Capital Corridors (Bowland and Otto, 2012). The MDC’s core focus is to rehabilitate an existing route by reviving the infrastructure, maximising investment, enhancing social development, and creating employment opportunities. It also focuses on advancing an inclusive and environmentally sustainable action system and facilitating anchor projects’ realisation (Mtegha et al., 2012). Both the South African and Mozambican governments supported the MDC. The Department of Transport runs and manages the MDC in both countries, but it is primarily the responsibility of the private sector to optimise the MDC. The MDCs cross-country linkages accelerate movement between areas and enable building and upgrading railways and roads and a border post between Mozambique and South Africa. The MDC has created jobs, contributed to GDP growth, and provided exports and investment opportunities in both countries (Söderbaum, 2001).

The MDC implementation process consisted of an initial set-up that involved procuring institutional and socio-economic data and the appointment of a project manager to manage the project at the conceptual stage. It also included a pre-feasibility phase led by a project manager, political and technical structural formation at various levels, feasibility study with crucial stakeholders, identifying and evaluating lead/anchor projects and the programme of action, and preparing a document setting out viable projects and investment opportunities. Afterwards, a formal presentation followed. The vision, objectives and opportunities, and anchor projects were presented and an exit strategy to “hand over” the project to structures such as the MCLI (Maputo Corridor Logistics Initiative, 2019).

The steel and aluminium plants that anchor the corridor’s eastern end makes the MDC more viable. The MDC has, however, also faced challenges. It has experienced fragmented community engagement and environmental, governance, and investment issues. One notable failure was the inability to set up a one-stop border post between South Africa and Mozambique. However, there have been upgrades to existing border posts to ease the transportation of goods (Cohen, 2008).

The MDC offers essential lessons. First, it emphasises the importance of state champions and support, which facilitates decision-making and implementation. There should, however, be a balance in this approach to prevent weak “heroes” from undermining the project. The MDC also highlights the importance of building formal and informal institutional capacities to fill any skill, the institutional gap that could arise, and the need to complete simple bureaucratic procedures.

Furthermore, the “fast-tracking approach” creates visible benefits and can spur a specific “multiplier effect” and a jumpstart to the economy. However, it can also prove counter-productive and negatively influence the management and the quality of investment projects. Therefore, it is necessary to ensure it is not yet another major project with little to no local links and to ensure that it relates with and considers the local realities on the ground. Again, the MDC emphasises the need to integrate all levels of government institutions in the corridor design. The MDC was heavily centralised with a focus on speed and large infrastructure projects. This situation was heightened by inadequate engagement with the local community (Bek and Taylor, 2001). This insufficient involvement of the affected local communities was not only on the South African side, illustrated by residents in the Matola area having lodged grievances regarding the government’s lack of consultation regarding the N4 toll road (Mtegha et al., 2012).

A further lesson to learn from the MDC is about environmental sustainability. Despite environmental sustainability being one of MDC’s four stated objectives, there was little focus on environmental sustainability. This objective was premature, given that the legislative tools to ensure environmental sustainability were not yet promulgated in South Africa or Mozambique at the time of the MDC in 1996. Therefore, it is essential to ensure an enabling regulatory environment exists, which requires thorough environmental impact assessments to construct corridor infrastructure (Mitchell, 1998). Furthermore, environmental considerations must be appraised holistically and not merely on a project basis. The project-based approach, where environmental concerns are considered on a project-by-project basis rather than on a holistic corridor basis, whilst necessary, must be accompanied by a holistic overview of the corridor impacts (Mitchell, 1998).

There must be an overarching “corridor” environmental strategy and assessment to ascertain the entire corridor’s effects. The absence of a bottom-up approach in the MDC evidences a “democratic deficit” in local participation. Lack of community participation can undermine the state’s role and present the country negatively before its citizens. The government could correct this by adopting the bottom-up strategy and ensuring the early participation of all stakeholders (Cohen, 2008). Coherent promotion of corridor content, the use of PPP, and an enabling policy and regulatory environment by both governments encouraged the MDC and led to its success (Mtegha et al., 2012).

### The Nacala and Zambezi Valley Corridors of Mozambique

Much like the MDC, the Nacala Corridor (NC) is based on a transport corridor for Zambia and Malawi to the Nacala port in Mozambique, which existed until the Mozambican civil war dislocated the transport links. The NC is an excellent example of a corridor reinvigorated by the financial investment from a mining company, Vale, to ensure transportation from an extractives anchor project. It also evidences the trade-off many governments may need to make when a third party heavily invests in a corridor project, namely, an integrated user concession. This occurs when one company is responsible for extraction and transport to market and requires separate ownership of the mines and the transport infrastructure. Vale purchased a majority share in the NC concession in Mozambique, despite the then government of Mozambique’s general position that railway concessions should be independent of mines (Maennling et al., 2014). Possible reasons for this relaxation and trade-off could be the complexity of the project crossing Malawi and the difficulty of finding an investor with the necessary financial backing willing to make the significant investment.

From a physical security standpoint, the NC traverses three different Mozambican provinces: Tete, Niassa, and Nampula, avoiding areas known to be the site of “deadly clashes” between two political parties, FRELIMO and RENAMO. Thus, avoiding the most conflict-prone provinces of northern and central Mozambique. This strategy illustrates the importance of a safe corridor route. For example, the Zambezi Valley Corridor (ZVDC) had security issues directly affecting the usage of the railway line, causing many mining companies to stop using the Sena railway line. The ZVDC also contains lessons for countries when entering into regional agreements. The Mozambiquan government largely governs and coordinates the ZVDC. However, the governments of Zambia and Malawi could have been partners in both the ZVDC and NC, as opposed to signing a tripartite agreement for the Nacala line, and indeed this would have been more beneficial for them. The partnership would have unlocked the export capacity within the countries, especially Malawi. Much of Malawi’s production costs are attributable to transportation and Malawi subsidies to commuters on the Nacala line. A partnership between the two countries would have substantially dropped their transportation costs.

## Applying the lessons to the Nigerian context

### Practicalities

The government and all stakeholders must take various practical steps for Nigeria to create and benefit from MRCs effectively. Practical steps are essential, such as developing an economic plan that integrates the MRC to actualise Nigeria’s objectives. An economic strategy is crucial in enabling and leveraging private sector investment through organised and prioritised investments across four dimensions—infrastructure, livelihoods, governance, and environmental and social impact. It also integrates mineral-related activities in specific resource-rich regions in Nigeria and sets the stage for economic diversification. The corridors we considered in the “Lessons from Resource Corridors in Mozambique” section prioritised these dimensions, and Nigeria can adopt a similar design, especially since the Mining Roadmap, 2016, lacks a corridor development plan.

From our contextual discussion in the “Lessons from Resource Corridors in Mozambique” section, the following lessons arise from MRC development for consideration in Nigeria’s planned MRC establishment.

### Infrastructural lessons

Infrastructure is key in the development of any corridor. As seen in the MDC, there was a focus on rehabilitating existing routes by reviving the infrastructure and building and upgrading railways and roads and a border post between Mozambique and South Africa. The lesson for Nigeria is that it must put in place the hard and soft infrastructure necessary for operating a MRC, such as railways and road networks or leverage on anchor projects. Fortunately, the Lagos-Ibadan-Kano-Maiduguri rail line is already under construction, with works currently at the Ibadan axis. New roads are under construction, and old ones such as the Lagos-Ibadan-Benin-Warri-Port Harcourt road are being rehabilitated.

Whilst these efforts are commendable, it is necessary to rehabilitate and construct new roads and connect resource-bearing communities in the ongoing infrastructural development. Some areas with mineral deposits require access roads and rail terminals to transport minerals from sites to processing locations or export to other places. It is necessary to increase the number of rail terminals along the rail line and make them commercially viable. This increase will give local communities access to mine sites and enable them to participate in the MRC activities and transport agricultural produce for an inclusive economy.

The power supply from the national grid is not where it should be now. Over 50 million homes currently jostle for power from less than 4,000 megawatts of electricity from the national grid (USAID, 2020). It means that alternative energy sources are necessary to bolster electricity supply to homes and industries to facilitate economic activities. Off-grid renewable energy sources such as solar power, wind farms, and hydropower dams can assist in power generation given high wind and sunshine in Nigeria.

Another infrastructural lesson is that anchor projects are essential. A such, the Ajaokuta and Aladja steel rolling mills need revitalisation to ensure steel production for onward transportation to areas where they are required. In addition, local utilisation of steel can reduce its importation and the demand for foreign exchange. There is evidence that the FGN has opened talks with a Russian firm to rehabilitate the Ajaokuta steel plant (Olurounbi, 2019). However, this talk needs to evolve beyond words if Nigeria hopes to create jobs and diversify its economy by processing and exporting steel.

### Social lessons

Developing a RC has social consequences as it sometimes results in community displacement, impact on livelihood, and possible uneven development (Integrated Resource Corridors Initiative, 2015). Laurance et al. (2015) observe that the decision to establish a MRC should continue if mitigation measures are in place. For Nigeria, the lesson is that a social impact assessment is essential to understand and reduce the likely impacts the corridor development will have on communities along the corridor path. If multiple corridors are proposed, a separate social impact assessment must be concluded for each one. It also implies that compensation is necessary to accommodate potential damages for community loss of livelihood and property. The evaluation and compensation scheme will enable communities to develop sustainably and participate in corridor activities.

Again, initiatives that will assist local people in blending into corridor activities are essential. Business and technical support and access to finance and risk-sharing schemes are necessary to upscale local capacities. Nigeria should consider launching pilot programmes to train people on MRC activities and create community awareness. A pilot scheme can start in the Ajaokuta area before being rolled out across the sector. Equivalent efforts to match the several skills development programmes to private sector requirements are also essential for job creation in project communities.

Support schemes linked to MRCs upscale agriculture and agribusiness and offer an opportunity for inclusive economic growth since the same MRC infrastructure can facilitate agricultural activities, agro-processing, and connect markets. Local farmers need access to roads to move their products just as artisanal miners need to transport extracted mineral resources. Though, a thorough review to understand the agricultural and agribusiness challenges is necessary.

### Environmental lessons

The development of a MRC raises issues around ecological and climate change concerns which can affect local communities (Adam Smith International, 2015). It also presents the need to preserve areas of high conservation value during road construction (Hobbs and Kumah, 2016). We gleaned from the “Lessons from Resource Corridors in Mozambique” section that despite the environmental focus of the MDC, it was poorly implemented because of the absence of a regulation to back it up. Nigeria is not a stranger to the effect which MRCs can have on the environment. The loss of farmland in the Jos Plateau due to the historic tin mining corridors (Onwuka et al., 2013) is still evident. However, the FGN can avoid the impact by ensuring adequate and extensive environmental impact assessments and enacting the proper environmental regulation early in the development process.

The importance of an existing environmental regulatory framework cannot be understated, mainly concerning climate change impacts. The last two decades have seen ecological and social assessments form part of funding decisions and the creation of frameworks such as the Equator Principles, which have guided the thinking around financing towards a more ecologically sustainable framework. A lesson learned from the MDC is that whilst its short-term approaches to growth characterise the SDI process, there was a balance between fast-tracking and streamlining processes. This impact would require the FGN to mainstream steps to mitigate carbon emission and protect local communities’ ecology and water sources since mining and corridor activities exist simultaneously. The absence of sustainable water use, for instance, will not be in the best interest of communities, especially in Northern Nigeria with rocky soil, which also requires water for irrigation purposes. Where communities face water challenges for irrigation purposes, designing artificial lakes may be necessary to meet that need.

Finally, land acquisition follows MRC development, the implication being that the regulatory regimes at both federal and state levels must consider the need for adequate compensation. There will also be a need to build capacity to verify land investments and strengthen land acquisition and the FGN’s ability to manage land acquisition related to MRCs. Again, it is essential to incorporate community benefits sharing into the process, requiring investors to execute CDAs with local communities and provide them with infrastructural support such as hospitals and schools.

### Governance lessons

The proper governance of a corridor is crucial to its effective operation (Söderbaum, 2018), with states having a major role to play (Söderbaum and Taylor, 2018). The MDC offers governance direction by using the now-defunct MCLI in managing the MDC, and Nigeria can adopt a similar strategy. Capacity building is essential at all corridor governance levels, especially in corridor planning and developing open and transparent communication among numerous stakeholders. It is also necessary to enable the management, planning, and implementation of the MRC. Government ministries and non-governmental organisations can play a part in the corridor governance, although they need the training to perform their function optimally and the law reformed.

Furthermore, security and community engagement are essential to corridor establishment. Nigeria has experience with security challenges. Due to the Boko Haram attacks in 2011, the government abandoned the rehabilitation of a trunk line from Borno to Maiduguri (Chen, 2018). This implies that security measures are essential to check the activities of armed bandits, terrorists, and extremist groups, especially in the Northern part of the country, to enable free movement of goods and services. This step is critical as terrorist groups can slow down a corridor’s progress, as seen in the China-Pakistan Economic Corridor. Again, community engagement is vital to achieving planned results in the development of RCs, as the absence of community engagement is a recipe for low corridor potentials.

## Recommendations and conclusion

This paper makes a case for a MRC in Nigeria and provides practical approaches to facilitate the successful development of the MRC. Relying on our evidence sources, analysis, and discussions, we recommend that Nigeria develop a framework strategic implementation plan for MRC operation and an inclusive budget together with an explicit message that only the best MRCs will receive funding. The government should appoint a project manager to realise and implement the strategic plan and corridor objectives from the conceptual phase. Again, environmental and social impact assessments should be carried out to determine the possible damage in the development process and compensation paid out to affected local communities. This assessment is essential as the construction and rehabilitation of roads and rail lines will impact community livelihood. As seen in Fig. 2, “Where will the MRC be?” section, most access roads and rail lines criss-cross communities and farmlands.

Nigeria needs to set and state the MRC parameters regarding funding and decide on the number of corridors and whether a MRC will be domestic or international. From the literature, PPPs as a funding mechanism are the dominant practice. A domestic MRC is recommended for Nigeria initially, given the absence of a clear geographical need for the MRC to connect international/regional boundaries. Again, the country’s size and the need to eliminate possible risks emanating from regional corridors favours a domestic corridor at this stage in Nigeria. A domestic corridor will assist Nigeria to build on its internal strengths, sustain the economy, prepare its institutions and governance structures, establish the relevant laws, and stabilise its infrastructural capacity before considering regional corridors. Finally, Nigeria’s current security challenges do not support a regional corridor. Insurgents, Boko Haram, bandits, and kidnappers along the corridor route will discourage other countries from active participation in the corridor activities, thus undermining the potential of the corridor.

At the right time, Nigeria should take advantage of existing regional synergies across the Gulf of Guinea through the ECOWAS network to connect with other countries, if desirable. Regional corridors are open to international crises, different regulatory regimes, external shocks, and other countries’ internal security risks. Due to Nigeria’s geographic position, there is no need for regional corridors to create the shortest route to a port, a likely option for many extractive operations. Suppose Nigeria wishes to create a regional corridor; it should ensure clarity where there is a gap or a difference in regulation in different countries. Parties should also enter into a written agreement to guide corridor activities. These agreements must cover the development of the corridor and the allocation of institutional and regulatory responsibilities. Issues such as border management, third party access to the corridor infrastructure, tariff, and pricing mechanisms and controls over the regulatory environment must be decided and reduced to writing. The various countries must show the political will to ensure uniformity in the corridor in their respective countries.

Pre-feasibility and feasibility studies particular to each proposed MRC should be put in place, with clear terms of reference for further assessment before selecting a corridor location. The corridor selection should be based on agreed criteria and practically help local communities benefit from corridor jobs and supply contracts. Again, Nigeria should consider identifying critical infrastructures, strengthening technical capacities and institutional structures and networks, and providing strong political and bureaucratic support. The establishment of the MDC was a function of existing resources and institutional capabilities. The varying stages of critical infrastructure, security situation, mineral clusters, and in-country end-use of mineral materials should also inform the evaluation for competing for MRC choices. Priority should be given to remedial actions for the corridors to progress with MRC satisfying these criteria.

It is necessary to secure anchor projects for the MRCs to create a multiplier effect and exploit Nigeria’s current infrastructural opportunities. The availability and suitability of anchor projects should also inform the choice of location for MRC. International support and partnership are indispensable, and the cooperation of all tiers of government in Nigeria is critical. Nigeria needs to establish a decision-making structure that “owns” the overall mandate and holds overall strategic responsibility, necessary for leadership, monitoring, and assurance, including the questions of integrity and good governance and the confidence of domestic and international stakeholders. It is also essential to ensure active community involvement and community and government “buy-in” at an early project stage. The absence of community engagement could prevent a corridor from achieving sustainable development outcomes, and it can also create disaffection in communities and spread grievances that can derail the project.

Nigeria must ensure that the various stakeholders collaborate, the absence of which could impede the potential of a MRC. A lack of collaboration will also duplicate efforts, waste resources, and promote enclave developments, resulting in opportunities that will not benefit all sectors and local communities. Further, accountability and transparency must be central to the core principles of the MRC’s governance structure to make the most of their contribution to sustained economic development and poverty reduction. Geographic data should be made available by the government to ensure adequate mapping of the corridor area. Having access to a suitable mapping of corridors, geological assets, legal boundaries, and natural boundaries in the same format and with the capacity to superimpose all this information onto one map would hugely facilitate the development of MRCs.

In conclusion, this article shows how Nigeria can diversify away from petroleum, boost the mining and agricultural sectors, and encourage commercial activities and industrialisation by developing a MRC. This idea requires a framework strategic implementation plan leveraging foreign and private investment, such as with the NC, where a mining company funded infrastructure rehabilitation (Rasagam et al., 2014). The development of MRCs in Nigeria can assist in the actualisation of the nation’s Mining Roadmap, 2016, especially in the realisation of the targeted 10% increase in the minerals sector’s contribution to the GDP by 2026 (MMSD, 2016). Further, there are several positive aspects of developing a MRC ranging from developing economic linkages (fiscal, production, and consumption), supporting the artisanal and small-scale miners, growth of the towns along the transport corridor, intersectoral development, and growth of the downstream mineral sector, among others.

Again, this research shows that the MRC provides a great opportunity to open up the country to national and regional growth and development. This can be seen from the regions connected by the Maputo Development Corridor (MDC), Mtwara Development Corridor Tanzania, the Lamu Port South Sudan Ethiopia Transport (LAPSSET) Corridor, the Nacala Corridor in Mozambique, for instance. However, the development of a MRC requires careful planning to be successful and achieve the desired outcomes. Nigeria can draw lessons from the successes and challenges of corridors within Mozambique in planning and implementing its MRC, as discussed in the “Lessons from Resource Corridors in Mozambique” section. This research has identified some key lessons Nigeria can learn from Mozambique in the “Applying the lessons to the Nigerian context” section, including social, environmental, and governance lessons in designing and planning the project. As such, there is a need for multi-stakeholder engagement. The success of any corridor depends on the political will and support of all levels of government. More so, developing a suitable national plan, in conjunction with civil society and communities, will enable Nigeria to leverage MRC to tap into its abundant mineral resources for economic diversification.
